# In‐line monitoring of protein concentration with MIR spectroscopy during UFDF

**DOI:** 10.1002/elsc.202200050

**Published:** 2022-12-23

**Authors:** Adrianna Milewska, Géraldine Baekelandt, Sarra Boutaieb, Vitalii Mozin, Andrew Falconbridge

**Affiliations:** ^1^ Alvotech Reykjavík Iceland; ^2^ IRUBIS GmbH München Germany

**Keywords:** bioprocessing, downstream bioprocessing, mid‐infrared spectroscopy, process analytical technology

## Abstract

Rapid increase of product titers in upstream processes has presented challenges for downstream processing, where purification costs increase linearly with the increase of the product yield. Hence, innovative solutions are becoming increasingly popular. Process Analytical Technology (PAT) tools, such as spectroscopic techniques, are on the rise due to their capacity to provide real‐time, precise analytics. This ensures consistent product quality and increased process understanding, as well as process control. Mid‐infrared spectroscopy (MIR) has emerged as a highly promising technique within recent years, owing to its ability to monitor several critical process parameters at the same time and unchallenging spectral analysis and data interpretation. For in‐line monitoring, Attenuated Total Reflectance—Fourier Transform Infrared Spectroscopy (ATR‐FTIR) is a method of choice, as it enables reliable measurements in a liquid environment, even though water absorption bands are present in the region of interest. Here, we present MIR spectroscopy as a monitoring tool of critical process parameters in ultrafiltration/diafiltration (UFDF). MIR spectrometer was integrated in the UFDF process in an in‐line fashion through a single‐use flow cell containing a single bounce silicon ATR crystal. The results indicate that the one‐point calibration algorithm applied to the MIR spectra, predicts highly accurate protein concentrations, as compared with validated offline analytical methods.

**Abbreviations**
AEX: Anion Exchange Chromatography
ATR: Attenuated Total Reflectance
CEX: Cation Exchange Chromatography
CHO: Chinese hamster ovary
CPPs: Critical Process Parameters
CQAs: Critical Quality Attributes
DF: Diafiltration
DSP: Downstream Processing
FTIR: Fourier Transform Infrared Spectroscopy
GMP: Good Manufacturing Practice
IgG2: Immunoglobulin G2
IRE: Internal Reflection Element
mAb: Monoclonal Antibody
MIR: Mid‐infrared spectroscopy
PAT: Process Analytical Technology
TFF: Tangential Flow Filtration
UF: Ultrafiltration
UFDF: Ultrafiltration/Diafiltration

## 1 INTRODUCTION

Over the last decade, spectroscopic techniques have been demonstrated to be powerful analytical tools for process development and manufacturing in the biopharmaceutical industry [1, 2, 3]. They allow continuous and simultaneous monitoring of critical process parameters (CPPs), in particular the concentrations of metabolites, nutrients, and other critical quality attributes (CQAs) [4] with the goal of ensuring product quality and enabling process control [5]. However, measuring critical parameters in the downstream process is still lax—up to 61% of participants in an Aspen survey indicated that downstream bioprocessing solutions needed to improve, compared to only 39% for improvement in upstream bioprocessing [6]. In downstream processing (DSP), speed and accuracy are critical [7]. Unlike cell culture processes, which take on average 2–3 weeks, a full DSP run can be achieved in 4 days. Individual chromatography steps last under 20 min, hence analytical techniques to monitor CQAs or CPPs need to be in quasi‐real time for accurate information about the process.

The downstream process includes a larger number of steps, which are required to purify a target product from cell culture broth [8]. Typically, it involves Protein A capture step followed by appropriate polishing steps; consisting of cation exchange chromatography (CEX) and anion exchange chromatography (AEX), which are used to remove host cell‐related impurities, as well as process and product related impurities [8, 9]. The final downstream bioprocess unit operations include nanofiltration and ultrafiltration/diafiltration step (UFDF). The main objective of UFDF is to increase the product concentration through volume reduction (UF) and buffer exchange to final formulation buffer (DF) [10]. Targeting high protein concentration at the UFDF step possess a series of challenges, such as volume exclusion and Donnan equilibrium effects [11, 12]. Final protein and excipient concentrations are based on either weight or theoretical calculations of the concentration factor. In a GMP environment, validated offline analytical methods are used to confirm these concentrations, which are critical for decision making [13]. However, offline analytics of CQAs require process pauses, adding significant time to the operation. In‐line monitoring tools allow for a solution, enabling non‐invasive, real‐time monitoring of multiple CQAs at the same time without the need for sampling, decreasing the risk of error‐prone dilutions and product contamination [14]. Recently, in‐line UV/Vis variable pathlength spectroscopy has gained a lot of attention for monitoring the downstream processes in real time for a broad dynamic range of concentrations [15]. Although the technology allows for fast and accurate concentration measurements and process control, it is feasible only for monitoring of a single component at a time, and it does not enable product quality monitoring [16]. Vibrational spectroscopies, however, have the potential to fill this gap, as they are able to elucidate the molecular and structural information about the sample in a non‐destructive way.

For instance, mid‐infrared Fourier transform spectroscopy (MIR‐FTIR), which is based on the interaction of molecules with electromagnetic radiation in the mid‐infrared range (400–4000 cm^−1^) [17], is able to monitor multiple parameters in real‐time. The molecular absorbance of mid‐infrared light causes rotations and vibration, which are classified by chemical functional groups [18, 19]. This selectivity can be applied in downstream processing to monitor protein and excipients concentrations, buffer exchange process, protein aggregation and overall product quality [20, 21]. Due, in part to the strong absorption of the water bands in the region of 1600 cm^−1^, which overlap with the protein amide bands in the spectra [22], MIR‐FTIR has often been underused. Modern MIR instruments, however, are equipped with an ATR accessory, which can mitigate this issue. ATR‐FTIR is a method where the effective pathlength is significantly reduced due to the high refractive index infrared‐transparent internal reflection element (IRE) [23, 24]. In ATR‐FTIR, the infrared radiation is directed at an angle of incidence larger than a critical angle (in most cases a 45° angle) causing internal reflection that creates an evanescent wave. When the sample is placed in close proximity to the IRE, only a thin layer adjacent to the surface is probed (1‐2 mm depth) through attenuated total reflection [25]. ATR crystals made of diamond are the most commonly used due to their high refractive index. Diamond crystals are expensive and are therefore unsuitable for single‐use applications necessary within the bioprocessing field. Alternatively, cost‐effective ATR silicon crystals can be used. These ATR crystals have micromechanical structuring on the underside, produced through MEMS processing, in which a single internal reflection can occur [26, 27]. Due to the thinness of the silicon ATR crystal (0.5 mm), the full spectral range, including the fingerprint region. can be accessed with this accessory. Additionally, the penetration depth is comparable to a single‐bounce diamond ATR at a 45° angle [26]. As the silicon ATR accessory used in this study has a comparable performance to a single bounce diamond ATR crystal, it presents itself as a perfect alternative for bioprocess applications, where single‐use solutions are often required.

Here, we present an in‐line solution for monitoring protein concentration during UFDF step of downstream purification process. The MIR spectrometer was connected to the UFDF setup through a single‐use flow cell comprising a single‐bounce, multiridge silicon ATR crystal (proprietary to IRUBIS GmBH). The protein concentration was determined by a simple one‐point calibration algorithm, based on the absorbance of the amide I and amide II peaks, contrary to complicated multivariate data analysis modeling.

## 2 MATERIALS AND METHODS

### 2.1 Materials

Monoclonal antibodies (mAbs) IgG2 (∼150 kDa, pI 8.7–9.1) derived from a Chinese hamster ovary (CHO) cell cultures (Alvotech, Reykjavík, Iceland) were used at a nanofiltration step of a typical downstream bioprocessing purification sequence. Equilibration buffer containing 40 mM Excipient I and 135 mM Excipient II at pH 6.0 was exchanged with diafiltration buffer containing 5 mM Excipient III and 240 mM Excipient IV at pH 6.0 serving as a final formulation buffer.

### 2.2 Equipment and experimental UFDF setup with MIR spectrometer

Monipa MIR spectrometer (IRUBIS GmbH, Munich, Germany) was connected in‐line via 3D‐printed BioMed Clear resin flow cell containing silicon ATR crystals (IRUBIS GmbH, Munich, Germany), directly through Male Luer Lock and MasterFlex L/S Precision Pump Tubing, Pharma Pure^®^, LS/14; 25 ft, internal diameter 1.6 mm (Cole–Parmer, Vernon Hills, IL, USA) on the feed line.

Four independent UFDF experiments were performed using a Repligen KrosFlo^®^ KR2i Tangential Flow Filtration (TFF) System and Repligen TangenX™ SIUS™ PDn 0.02 m^2^ (LP) HyS 30 kD single‐use filtration cassettes. The experiments were conducted at a membrane loading between 500 and 700 g/m^2^ of protein. The cassettes were installed with a torque setting between 14.0 and 20.0 Nm using the provided gaskets to prevent leakage. The cassettes were preconditioned with 10 L/m^2^ water flush followed by a 10 L/m^2^ equilibration buffer flush (40 mM Excipient I, 135 mM Excipient II, pH 6.0). For all four UFDF runs, the starting protein solution was at a concentration of 17 mg/ml, concentrated to 40 mg/ml during the first ultrafiltration step (UF1). This was followed by diafiltration (DF) with the final formulation buffer (5 mM Excipient III, 240 mM Excipient IV, pH 6.0) for seven diavolumes before up‐concentrating during the second ultrafiltration step (UF2) to concentrations between 90 and 200 mg/ml, varying between runs to cover a wide range of concentrations. The holdup volume in the system was determined to be 14 ml, in which 0.6 ml is the dead volume of the flow cell connected on the feed line. All trials were performed at a constant transmembrane pressure, typically 1 bar and feed flow rates around 60 ml/min. Constant transmembrane pressure was attained using the retentate valve control set‐up manually.

Samples for offline analysis with OD280 were collected throughout the four UFDF processes, including samples after UF1 and UF2 as well as intermediate time points during the second concentration step in one of the runs (Tables S1‐S4).

### 2.3 MIR measurements

**FIGURE 1:**
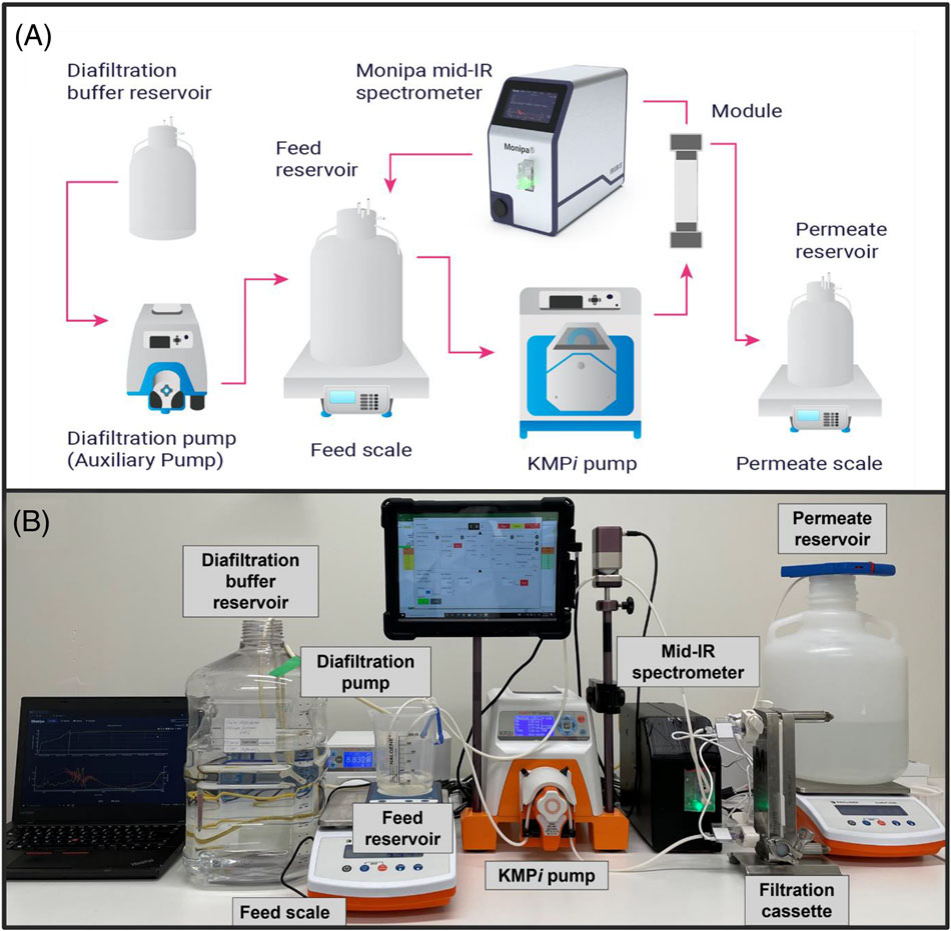
(A) Schematic representation of MIR spectrometer (Monipa 1, IRUBIS GmbH) connected to the feed line of the Repligen KrosFlo KR2i TFF system. (B) Laboratory set‐up of MIR spectrometer connected to the Repligen KrosFlo KR2i TFF system.

For these measurements, Monipa, the proprietary FTIR spectrometer (IRUBIS GmbH, Munich, Germany) was used. It has a resolution of four wavenumbers, which generates a MIR spectra every .5 s. Spectra shown on the instrument are averaged over 60 s, for optimal signal to noise ratio. An attachable flow cell with integrated ATR crystal (proprietary to IRUBIS GmbH) in single bounce mode, allows for fluid flow‐through. Hence the Monipa can be placed directly in‐line with the Repligen KrosFlo KR2i TFF system, as shown in Figure 1.

From the MIR range, a variety of different molecules can be identified, which is especially useful in a complex environment such as bioprocessing. Protein peaks are shown in the region of 1450–1580 cm^−1^ and 1600–1700 cm^−1^ for amide II and amide I peaks, respectively. In addition to monitoring the protein peaks for the specific antibody in this study, the stabilizing agents can also be monitored, as excipients such as sorbitol, mannitol, and other sugars can be found from 995–1056 cm^−1^, well within the MIR range.

Raw MIR spectra were pre‐processed using baseline correction and smoothed with Savitzky–Golay filter in OriginLab Pro 2022 software. The coefficient of determination (R^2^) was calculated using linear fit and regression analysis in OriginLab Pro 2022 software, indicating the difference between the reference and predicted values.

## 3 RESULTS

**FIGURE 2:**
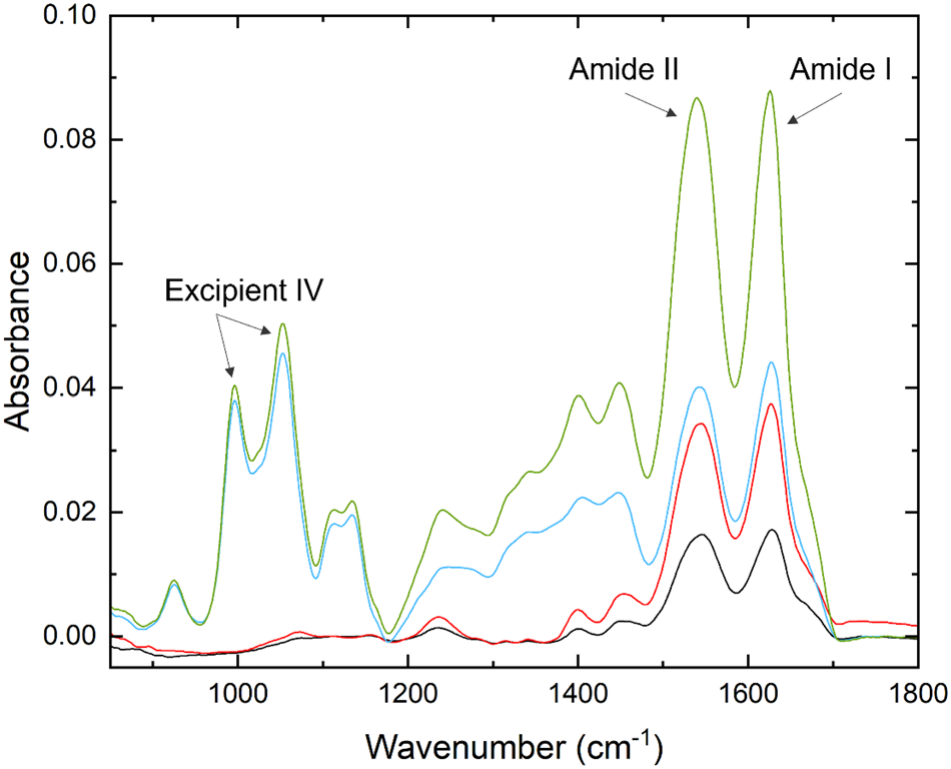
Representative MIR spectra of in‐process protein and excipients from selected timepoints during UFDF run (black trend—nanofiltrate, red trend—first concentration step, blue trend—after diafiltration, green trend—second concentration step).

In this work, the MIR spectrometer Monipa was connected directly to the Repligen KrosFlo UFDF setup via flow cell containing silicon ATR crystal to enable real‐time monitoring of the UFDF process. The spectra were averaged over 60 s at a resolution of four wavenumbers, yielding large amount of spectral data from four UFDF runs (Figures S1‐S8). The present technology allows for automated data piping and translation from the instrument to the software, enabling real‐time display of the predicted concentration values. To build predictive models of protein concentration, two most prominent spectral regions of amide I and amide II were selected. The most characteristic fingerprint region of protein secondary structure is amide I, found between 1600 and 1700 cm^−1^, which is derived from the C = O stretching vibration of the amide group tied to in‐phase bending of the N‐H bond and stretching of the C‐N bond. Another sensitive band region of proteins, amide II, can be found between 1450 and 1580 cm^−1^ and originates from in‐plane N‐H bending and the C‐N stretching vibration [23, 28, 29]. The representative spectra from different steps of UFDF process are displayed in Figure 2 below. The lowest spectrum (black trend line) represents the load material (nanofiltrate) containing the protein sample (around 17 mg/ml) in the equilibration buffer containing 40 mM Excipient I and 135 mM Excipient II, which traces are not displayed. The red trend represents the spectrum of the protein sample after the first concentration step to 40 mg/ml. The blue trend is a spectrum recorded after seven diafiltration steps, indicating full buffer exchange from equilibration buffer to final formulation buffer containing 5 mM Excipient III and 240 mM Excipient IV. The fingerprint region for Excipient IV has well‐defined maxima located at 995 and 1056 cm^1^, which are characteristic vibrations of common disaccharides, assigned to C = C bending and C‐O stretching, respectively [30]. Finally, the green trend represents the fully formulated drug substance at the target concentration after second ultrafiltration step with well‐defined amide I and amide II peaks.

**FIGURE 3:**
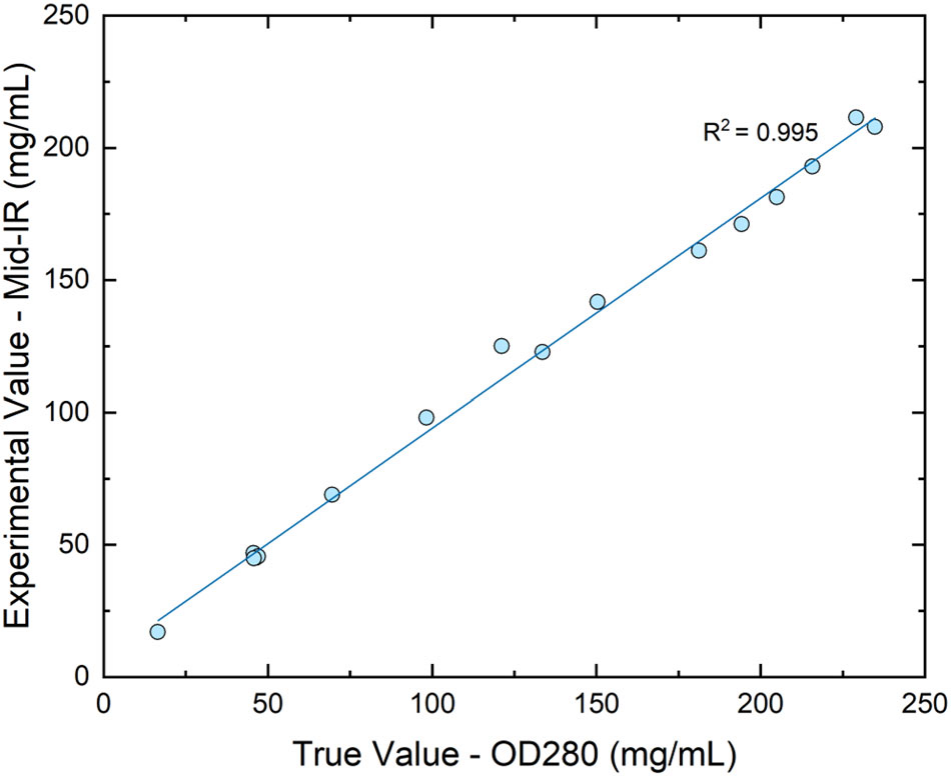
Linear regression model for protein concentration. X‐axis denotes the reference validated offline analytical assay OD280 and Y‐axis represents MIR predicted values. Coefficient of determination (R^2^) = 0.995.

According to Beer‐Lambert's law, the absorption is directly proportional to concentration [31], therefore the protein concentration can be determined from the MIR spectra based on the intensity of the amide peaks. The one‐point calibration algorithm developed by IRUBIS can predict the protein concentration with high accuracy, as compared to the validated offline reference method OD280 (Figure 3 and Tables S1‐S4). At higher concentrations, however, the variations between in‐line and offline measurements are more prominent. The disparities are the largest during the second ultrafiltration step in the UFDF process, where the concentration changes rapidly. In order to demonstrate the accuracy of the predictions, the final concentration in each run was different (ranging from 90 to over 200 mg/ml) to cover a wider range of concentrations. Furthermore, at this step, the protein concentration measured in real‐time in the flow cell might be slightly different to what is being drawn out of the retentate vessel at the same timepoint (see Figure 2 for the location of the flow cell and the retentate vessel). Nonetheless, the linear regression model for protein concentration suggests miniscule differences between predicted and true values, as the calculated coefficient of determination (R^2^) is 0.995, indicating high level of assurance of MIR measurements in real‐time.

## 4 CONCLUSIONS

In conclusion, we monitored protein concentration inline using a MIR spectroscopy during the ultrafiltration/diafiltration (UFDF) process in real time. The implementation of customizable, single‐use flow cells containing a silicon ATR‐crystal enables high sterility and monitoring of aqueous solutions. The protein concentration values are highly accurate as compared to validated offline reference methods. At higher protein concentrations (above 200 mg/ml) the variability between the MIR method and OD280 assay are larger, however, the one‐point calibration algorithm predicts the real‐time concentration with high precision, paving a way toward non‐invasive process monitoring and real‐time‐release.

## CONFLICT OF INTEREST

All authors declare that they have no conflicts of interest.

## Supporting information

Supporting Information

## Data Availability

The data that support the findings of this study are available from the corresponding author upon reasonable request.
