# Supplementary material for: In‐line monitoring of protein concentration with MIR spectroscopy during UFDF
Source: Eng Life Sci. 2022 Dec 23;23(2):e2200050. doi: 10.1002/elsc.202200050 (PMC9893749; doi:10.1002/elsc.202200050)
Supplement: Supplementary file 1 — Supporting Information [file ELSC-23-e2200050-s001.docx]

Supplementary Information

**Table S1.** Protein concentration measurements of UFDF samples from run 1 determined by OD280 and in-line IR measurements.

| **Sample name** | **Concentration** | |
| --- | --- | --- |
|  | OD280 (mg/mL) | Mid-IR (mg/mL) |
| **Nanofiltrate** | 16.5 | 16.98 |
| **UF1** | 46.3 | 45.04 |
| **UF2** | 98.3 | 98.11 |


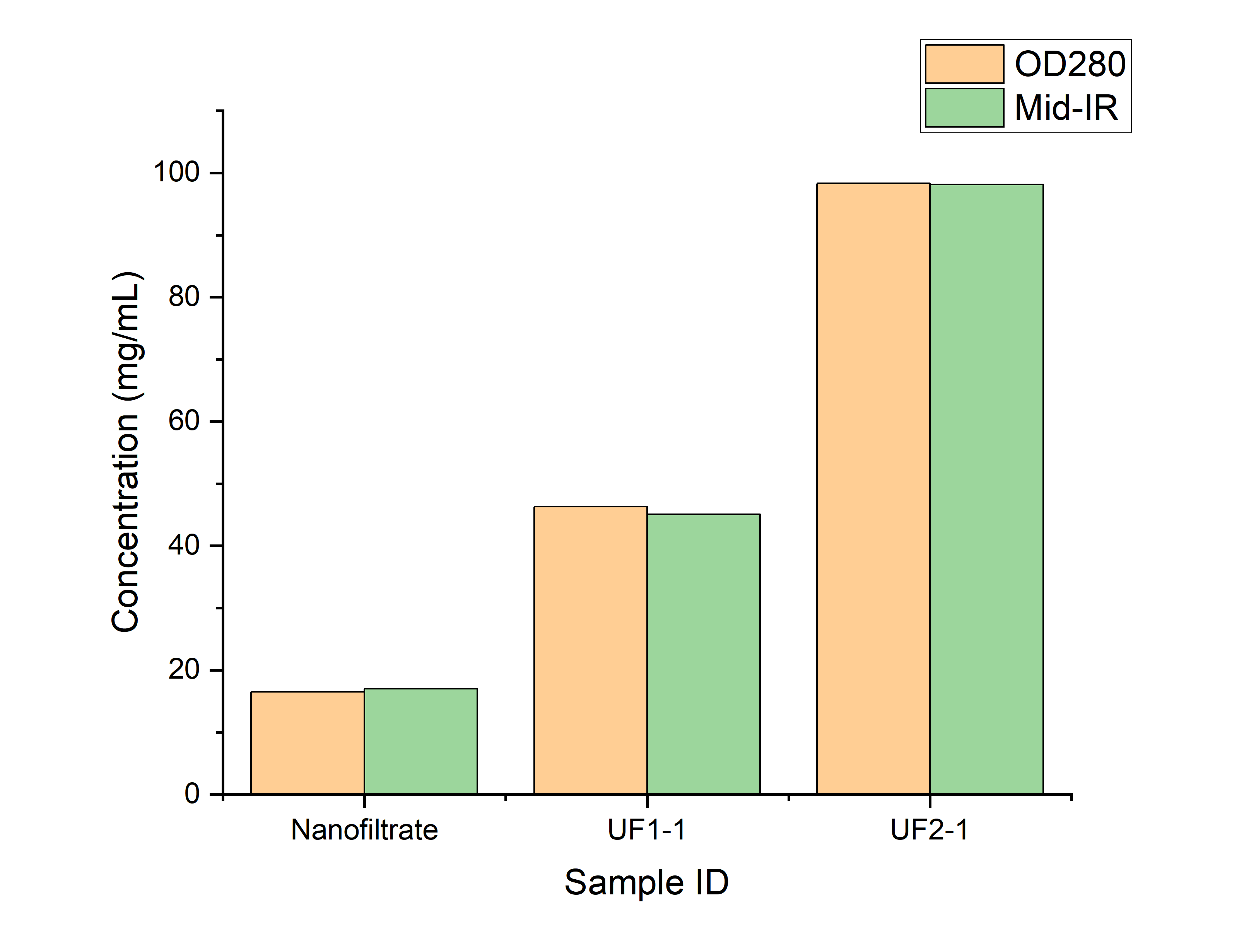


1. Bar plot – protein concentration measured in-line by Mid-IR spectrometer and offline reference method OD280 (UFDF run 1).


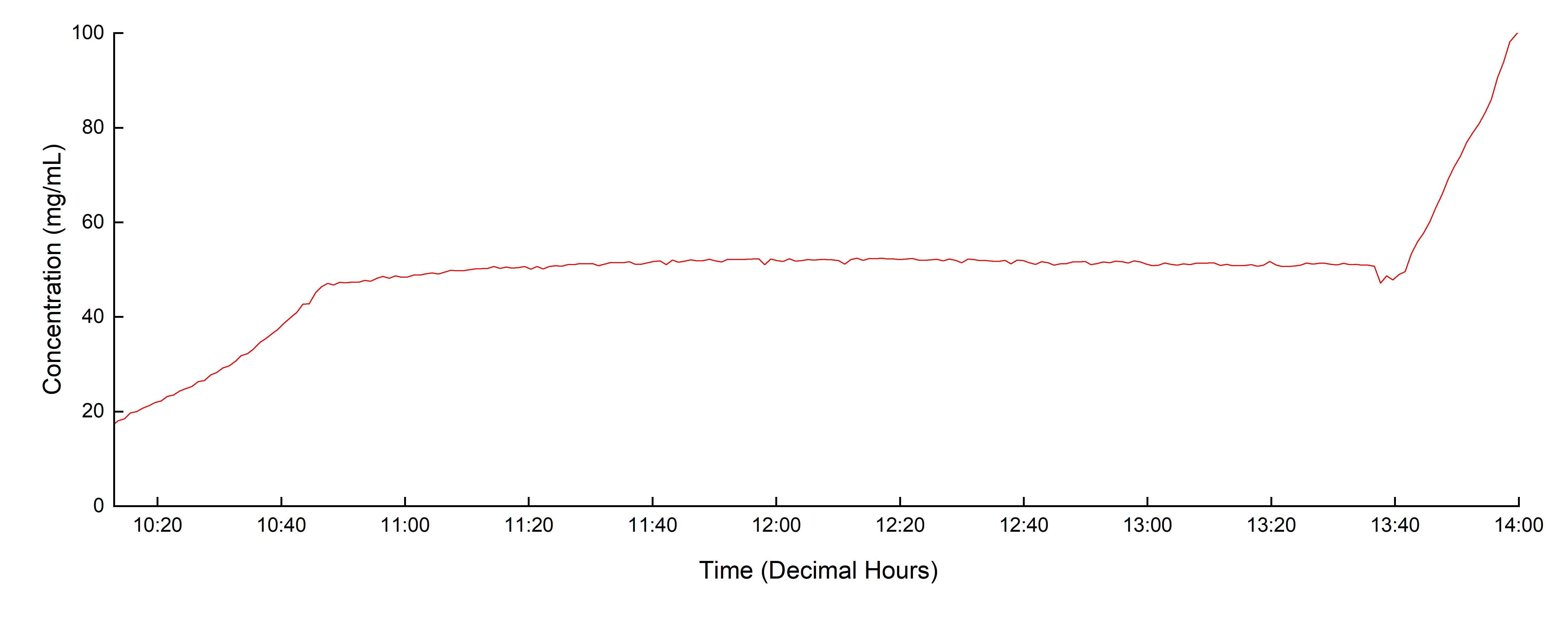


1. UFDF run 1 – real-time protein concentration trend

**Table S2.** Protein concentration measurements of UFDF samples from run 2 determined by OD280 and in-line IR measurements.

| **Sample name** | **Concentration** | |
| --- | --- | --- |
|  | OD280 (mg/mL) | Mid-IR (mg/mL) |
| **Nanofiltrate** | 16.5 | 17.095 |
| **UF1** | 45.7 | 46.82 |
| **UF2** | 121.2 | 124.96 |


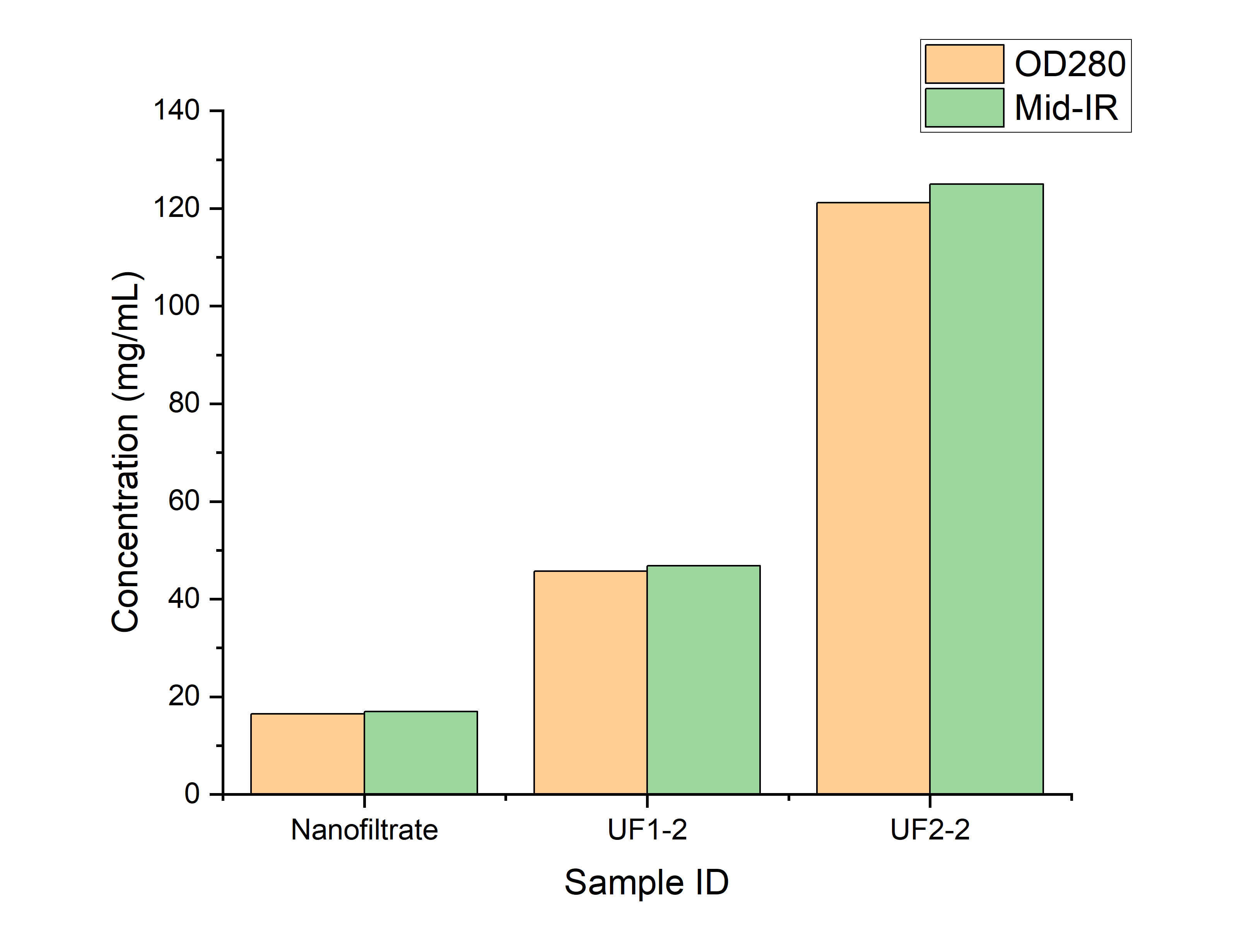


1. Bar plot – protein concentration measured in-line by Mid-IR spectrometer and offline reference methods OD280 (UFDF run 2).


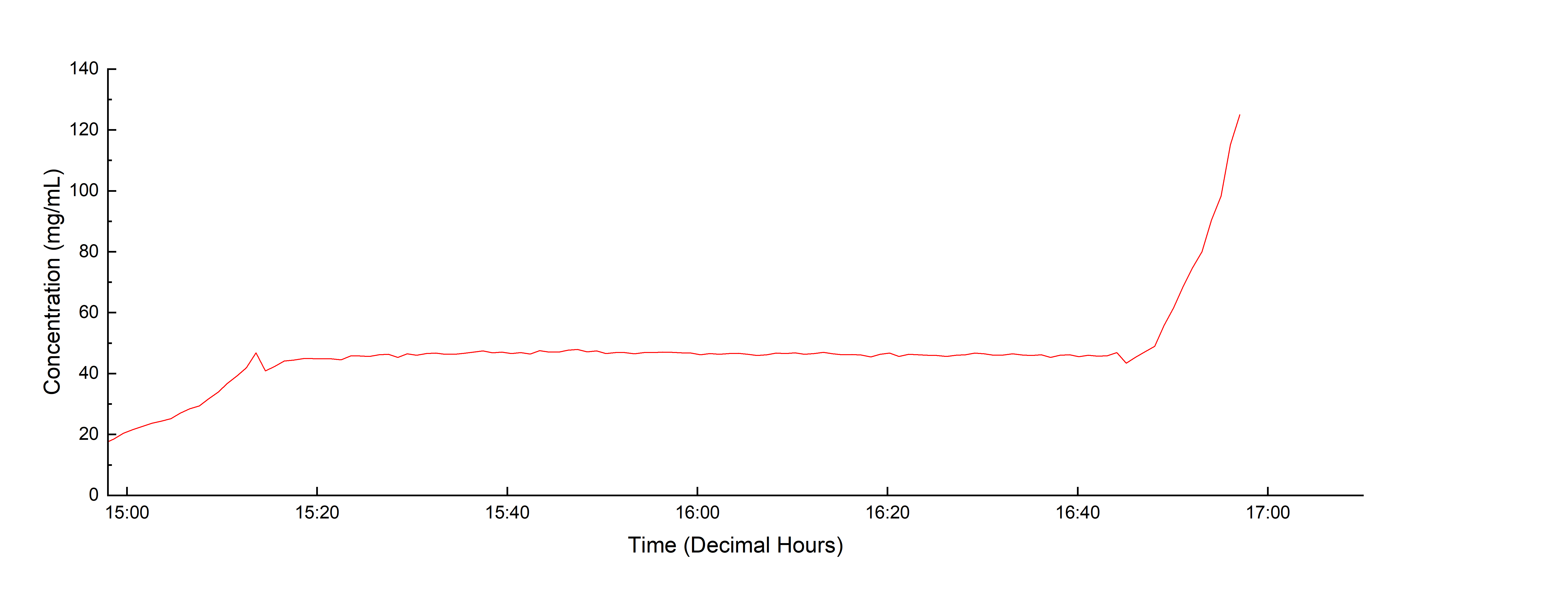


1. UFDF run 2 – real-time protein concentration trend

**Table S3.** Protein concentration measurements of UFDF samples from run 3 determined by OD280 and in-line IR measurements.

| **Sample name** | **Concentration** | |
| --- | --- | --- |
|  | OD280 (mg/mL) | Mid-IR (mg/mL) |
| **Nanofiltrate** | 16.5 | 17.47 |
| **UF1** | 47.1 | 45.56 |
| **UF2** | 234.8 | 208.002 |


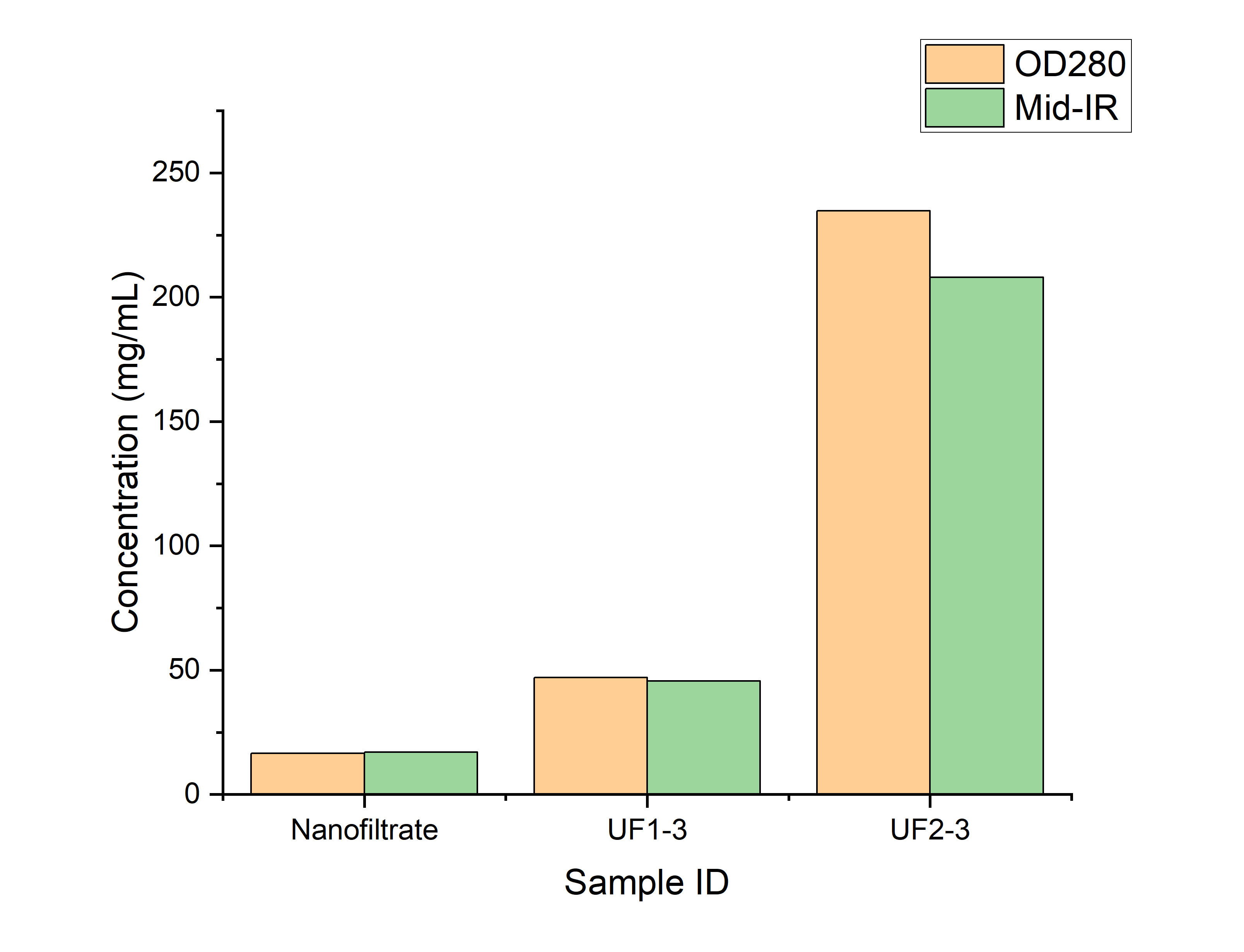


1. Bar plot – protein concentration measured in-line by Mid-IR spectrometer and offline reference method OD280 (UFDF run 3).


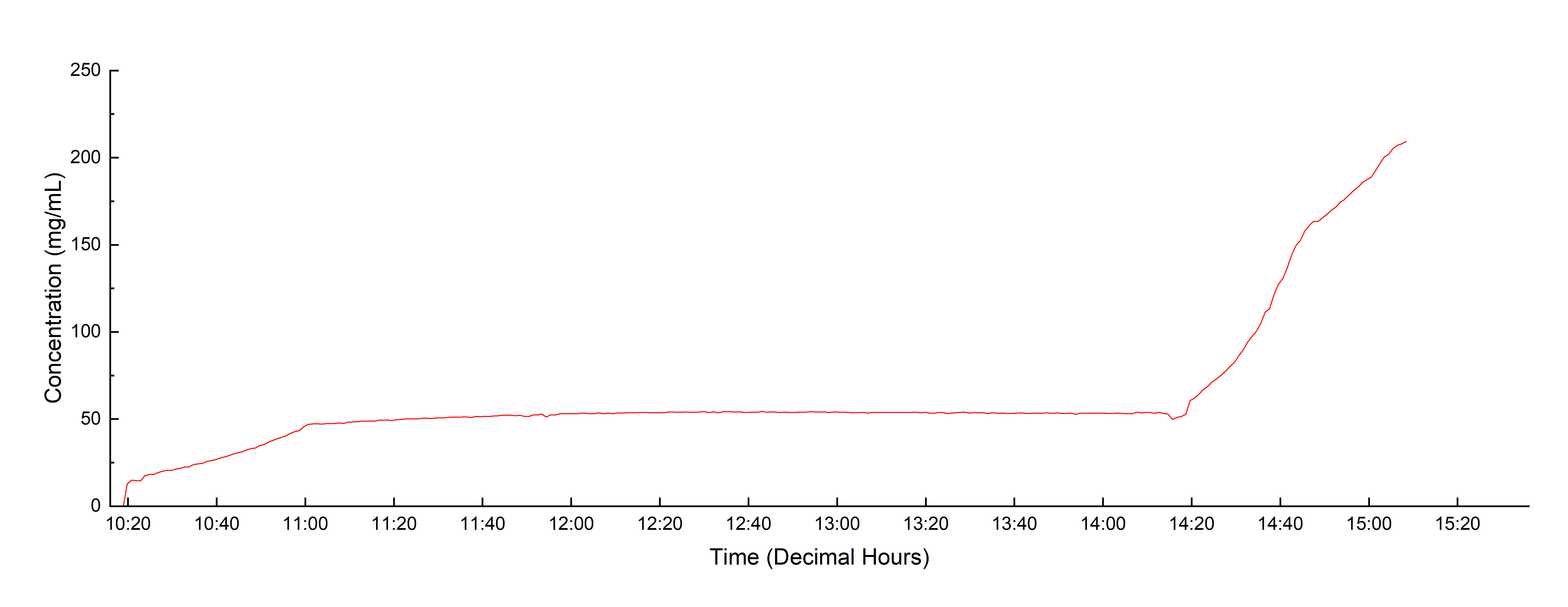


1. UFDF run 3 – real-time protein concentration trend

**Table S4.** Protein concentration measurements of UFDF samples from run 4 determined by OD280 and in-line IR measurements.

| **Sample name** | **Concentration** | |
| --- | --- | --- |
|  | OD280 (mg/mL) | Mid-IR (mg/mL) |
| **Nanofiltrate** | 16.5 | 17.237 |
| **UF1** | 45.8 | 44.908 |
| **A** | 69.6 | 69.01 |
| **B** | 133.6 | 122.67 |
| **C** | 150.3 | 141.66 |
| **D** | 181.2 | 161.09 |
| **E** | 194.2 | 171.08 |
| **F** | 204.9 | 181.32 |
| **G** | 215.7 | 192.89 |
| **UF2** | 229.1 | 211.501 |


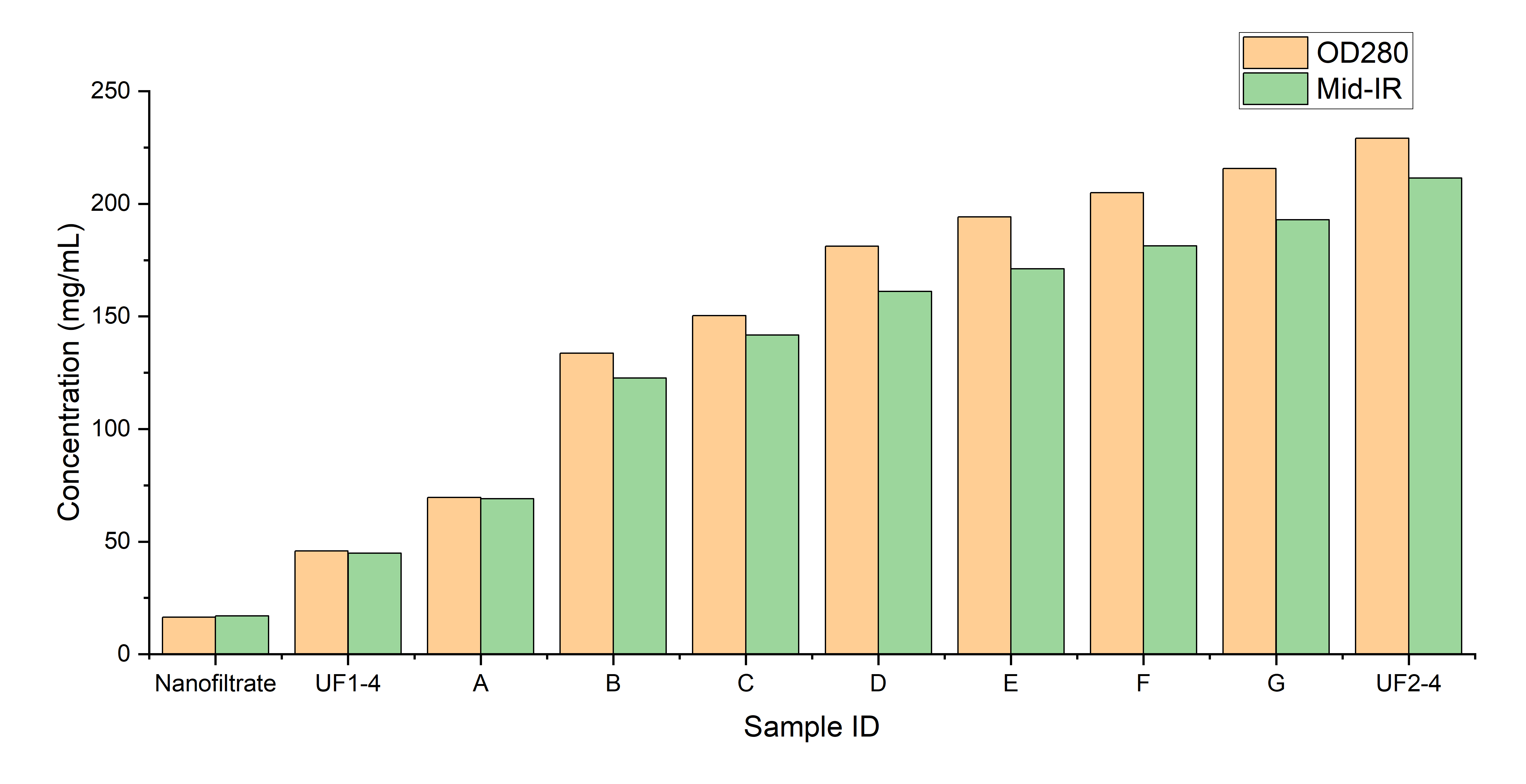


1. Bar plot – protein concentration measured in-line by Mid-IR spectrometer and offline reference method OD280 (UFDF run 4). Samples A-G represent intermediate steps during second ultrafiltration.


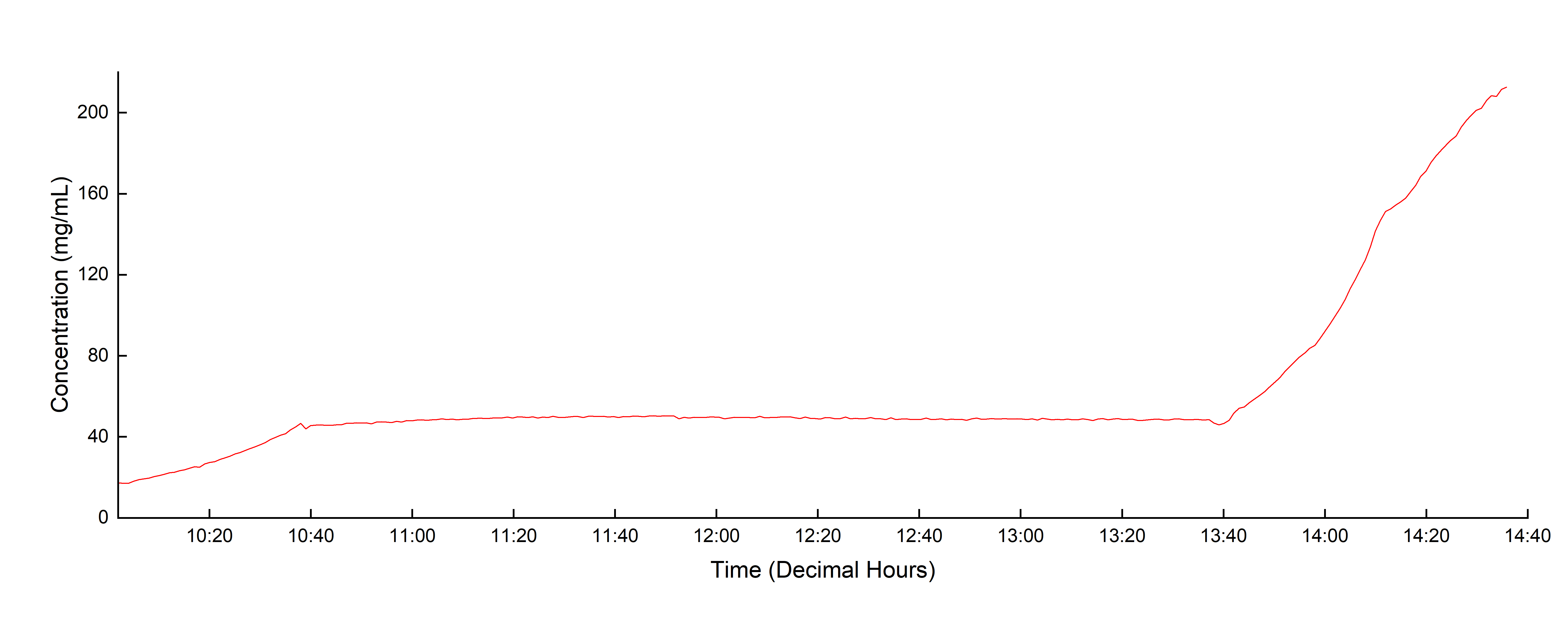


1. UFDF run 4 – real-time protein concentration trend
